# RasGRP2 inhibits glyceraldehyde-derived toxic advanced glycation end-products from inducing permeability in vascular endothelial cells

**DOI:** 10.1038/s41598-021-82619-0

**Published:** 2021-02-03

**Authors:** Jun-ichi Takino, Takuma Sato, Takumi Kanetaka, Kasumi Okihara, Kentaro Nagamine, Masayoshi Takeuchi, Takamitsu Hori

**Affiliations:** 1 Faculty of Pharmaceutical Sciences, Hiroshima International University, Hiroshima, Japan; 2 Faculty of Health Sciences, Hiroshima International University, Hiroshima, Japan; 3 Department of Advanced Medicine, Kanazawa Medical University, Ishikawa, Japan

**Keywords:** Cell biology, Molecular biology

## Abstract

Advanced glycation end-products (AGEs) are formed by the non-enzymatic reaction of sugars and proteins. Among the AGEs, glyceraldehyde-derived toxic AGEs (TAGE) are associated with various diseases, including diabetic complications such as diabetic retinopathy (DR). The risk of developing DR is strongly associated with poor glycemic control, which causes AGE accumulation and increases AGE-induced vascular permeability. We previously reported that Ras guanyl nucleotide releasing protein 2 (RasGRP2), which activates small G proteins, may play an essential role in the cell response to toxicity when exposed to various factors. However, it is not known whether RasGRP2 prevents the adverse effects of TAGE in vascular endothelial cells. This study observed that TAGE enhanced vascular permeability by disrupting adherens junctions and tight junctions via complex signaling, such as ROS and non-ROS pathways. In particular, RasGRP2 protected adherens junction disruption, thereby suppressing vascular hyper-permeability. These results indicate that RasGRP2 is an essential protective factor of vascular permeability and may help develop novel therapeutic strategies for AGE-induced DR.

## Introduction

Advanced glycation end-products (AGEs) are produced by the non-enzymatic reactions of carbonyl compounds, often of a sugar, and the amino groups of proteins, in the Maillard reaction^1^. Among the various AGEs produced in vivo*,* contingent on the type of carbonyl compound, there is evidence that glyceraldehyde-derived toxic AGEs (TAGE) are associated with diabetic complications, as well as insulin resistance, cardiovascular diseases, Alzheimer’s disease, hypertension, nonalcoholic steatohepatitis, obesity, and cancer^2–10^. In particular, the prevalence of diabetes is increasing year on year and is expected to reach approximately 700 million people worldwide by 2045^11^. Diabetic retinopathy (DR) is the leading cause of blindness in middle-aged and elderly people worldwide. It is the most common microvascular complication, affecting a third of diabetic patients. DR-onset risk is higher in type 1 diabetic patients, compared to type 2, and is strongly associated with long-term diabetic conditions and poor glycemic and blood pressure control^12^. The early stage of DR is characterized by abnormal vascular permeability and/or the formation of microaneurysms^13^. Therefore, increased vascular permeability, which induces the disruption of the blood-retinal barrier, is an important event for onset. A key cause of this is the accumulation of AGEs^14–16^. However, no drug has been approved to inhibit the accumulation of AGEs and the mechanism of TAGE-induced vascular permeability is not well understood.

We previously identified Xenopus *ras guanyl nucleotide releasing protein 2* (*rasgrp2*)*,* a homolog of the human *rasgrp2*, as a novel vascular related gene in Xenopus embryos^17–20^. The RasGRP2 protein, or GDP/GTP exchange factor (GEF)—part of the RasGRP family (RasGRP1-4)—activates small G proteins, such as Rap1^21^. In recent studies, we showed that RasGRP2 is expressed in vascular endothelial cells^22^. We also demonstrated that Rap1, activated by RasGRP2, suppresses tumor necrosis factor-α-induced apoptosis by suppressing reactive oxygen species (ROS) production, via NADPH oxidase (NOX) inhibition^23^. Furthermore, we discovered that RasGRP2 promotes the translocation of hexokinase-2 to mitochondria, via the R-Ras-phosphoinositide 3-kinase (PI3K)-Akt signaling pathway, and the suppression of apoptosis, by Bax translocation inhibition, via its signaling pathway^24^. These findings indicate that RasGRP2 may play an important role in the health of vascular endothelial cells, when exposed to various factors.

In this study, we report the mechanisms of vascular permeability induced by TAGE and the possibility it may act as a protective factor against vascular permeability in the vascular endothelium.

## Results

### RasGRP2 does not affect barrier formation

**Figure 1:**
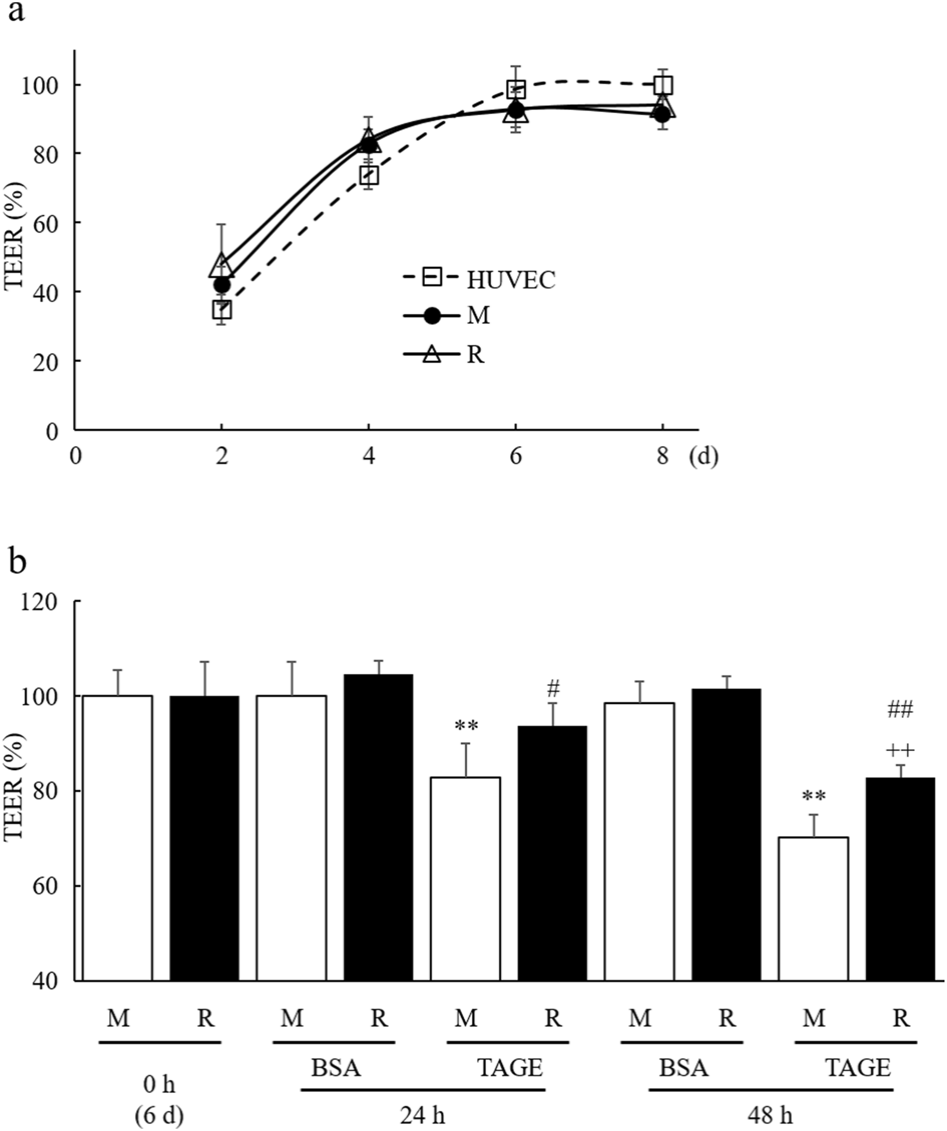
Barrier formation and suppression of TAGE-induced vascular hyper-permeability by RasGRP2. TEER value was measured using the Millicell-ERS. (**a**) Time-dependent change on barrier formation. The TEER value after 8 days for HUVEC was set to 100%. Square: HUVEC, circle: M cells, triangle: R cells. (**b**) Time-dependent change after BSA or TAGE treatment. The TEER value after 6 days for M cells was set to 100%. *M* mock cells, *R* RasGRP2-stable overexpression cells. Data shown as the mean ± SD (n = 3), **P < 0.01 compared with M cells of 0 h, ^++^*P* < 0.01 compared with R cells of 0 h, and ^#^*P* < 0.05, ^##^*P* < 0.01 compared with each TAGE-treated M cells.

To examine the influence of RasGRP2 on barrier formation in a cell membrane, we investigated the time-dependent change of the trans-endothelial electrical resistance (TEER) value, using Millicell-ERS. The TEER value for the Mock (M) and RasGRP2-stable-expression (R) cells reached a plateau at 6 days, similar to the human umbilical vein endothelial cells (HUVEC), with no significant difference between M and R cells (Fig. 1a). This result suggested that RasGRP2 does not affect barrier formation in the cell membrane.

### RasGRP2 suppresses TAGE-induced increase in vascular permeability

**Figure 2:**
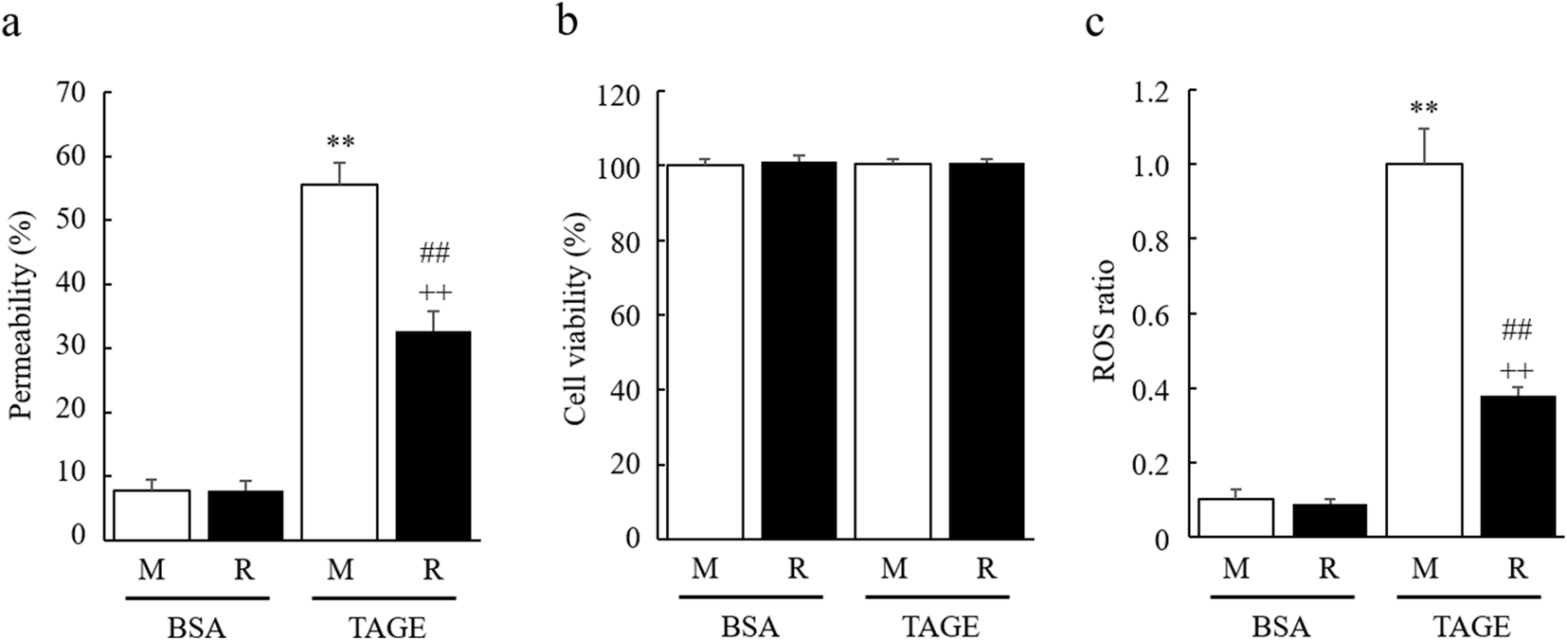
Reduction of TAGE-induced vascular hyper-permeability and ROS production by RasGRP2. (**a**) Permeability was measured using FITC-Dextran. (**b**) Cell viability was determined by Cell Counting Kit-8 assay. (**c**) Intracellular ROS was determined by CellROX Green. *M* mock cells, *R* RasGRP2-stable overexpression cells. Data shown as the mean ± SD (n = 3), **P < 0.01 compared with BSA-treated M cells, ^++^*P* < 0.01 compared with BSA-treated R cells, and ^##^*P* < 0.01 compared with TAGE-treated M cells.

We next examined the effect of TAGE treatment on the time-dependent change in TEER value. Cells treated with TAGE showed decreased TEER value over time, whereas both M and R control cells, treated with bovine serum albumin (BSA), showed no reduction. The degree of reduction was lower in R cells than in M cells (Fig. 1b). Similarly, when using fluorescein isothiocyanate (FITC)-dextran in detail, TAGE-treated R cells showed significantly lower permeability compared to TAGE-treated M cells (Fig. 2a). At this time, cell viability was not changed by TAGE treatment in either cell group (Fig. 2b). Furthermore, intracellular ROS levels were significantly increased by TAGE treatment in both M and R cells, although the increase was significantly lower in R cells (Fig. 2c).

Similarly, HUVEC also showed a significant increase in vascular permeability after TAGE treatment (Fig. S1) and showed the same behavior as M cells. Thus, immortalized HUVEC had similar barrier formation and vascular permeability performance to HUVEC, indicating that these M and R cells were unaffected by the transfection process.

### RasGRP2 inhibits vascular hyper-permeability by suppressing TAGE-induced ROS and non-ROS pathways

To investigate the effect of RasGRP2 on TAGE-induced hyper-permeability in vascular endothelial cells, we compared the ROS production and vascular permeability of M and R cells using N-acetyl cysteine (NAC), a ROS scavenger, diphenyleneiodonium (DPI), apocynin, an inhibitor of NOX, and LY294002 (LY), an inhibitor of the PI3K-Akt pathway.

**Figure 3:**
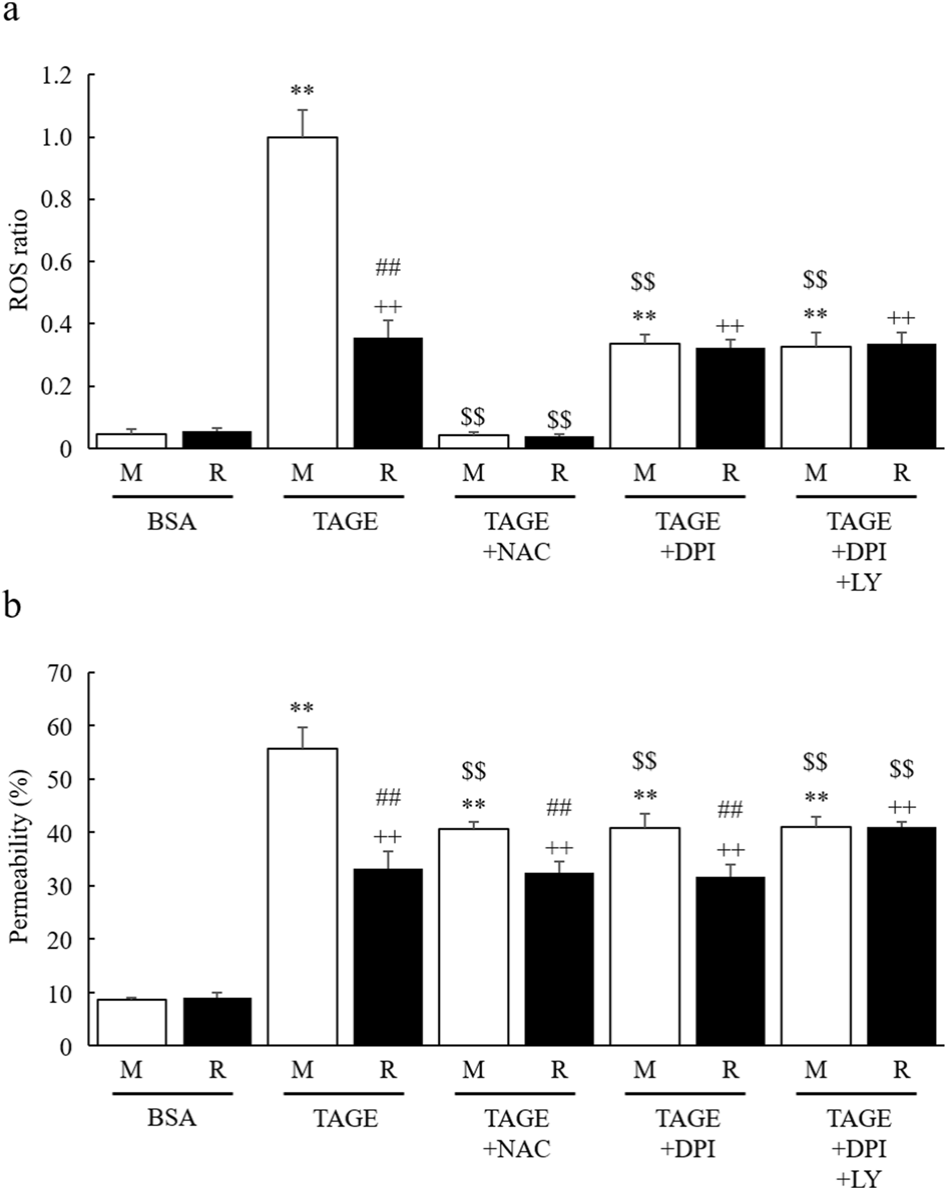
Reduction of TAGE-induced vascular hyper-permeability via ROS and non-ROS pathways by RasGRP2. NAC, a ROS scavenger, DPI, an inhibitor of NOX, and LY, an inhibitor of the PI3K-Akt pathway, were used to inhibit TAGE-induced ROS production, TAGE-induced ROS production via NOX and RasGRP2-induced Akt activation, respectively. (**a**) Intracellular ROS was determined by CellROX Green. (**b**) Permeability was measured using FITC-Dextran. *M* mock cells, *R* RasGRP2-stable overexpression cells, *NAC* N-acetyl cysteine, *DPI* diphenyleneiodonium, *LY* LY294002. Data shown as the mean ± SD (n = 3), **P < 0.01 compared with BSA-treated M cells, ^++^*P* < 0.01 compared with BSA-treated R cells, ^##^*P* < 0.01 compared with each TAGE-treated M cells, and ^$$^*P* < 0.01 compared with TAGE-treated cells alone.

In both M and R cells, TAGE-induced ROS was eliminated by NAC pretreatment. In contrast, TAGE-induced ROS was significantly reduced by DPI pretreatment in M cells, but not in R cells, resulting in a comparable ROS ratio (Fig. 3a). Similar results were obtained for apocynin pretreatment (Fig. S2). Furthermore, DPI + LY co-treatment did not alter TAGE-induced ROS in M and R cells (Fig. 3a).

In M cells, TAGE-induced hyper-permeability was significantly reduced by NAC and DPI pretreatments. However, there was no significant difference between NAC + TAGE and DPI + TAGE pretreatment effects. Neither pretreatment altered the magnitude of TAGE-induced hyper-permeability in R cells. In contrast, DPI + LY co-pretreatment did not alter the magnitude of TAGE-induced hyper-permeability in M cells but significantly increased the effects of TAGE in R cells (Fig. 3b). Similar results were obtained for LY pretreatment alone (Fig. S3). These results indicate that ROS is involved in TAGE-induced hyper-permeability and that the ROS pathway related to vascular permeability is dependent on NOX alone. In other words, NOX-independent ROS production by TAGE does not affect vascular permeability. Also, it indicates that RasGRP2 completely suppresses the ROS pathway related to vascular permeability and acts on some non-ROS pathways through Akt activation.

### RasGRP2 protects TAGE-induced perturbation of vascular endothelial-cadherin protein

**Figure 4:**
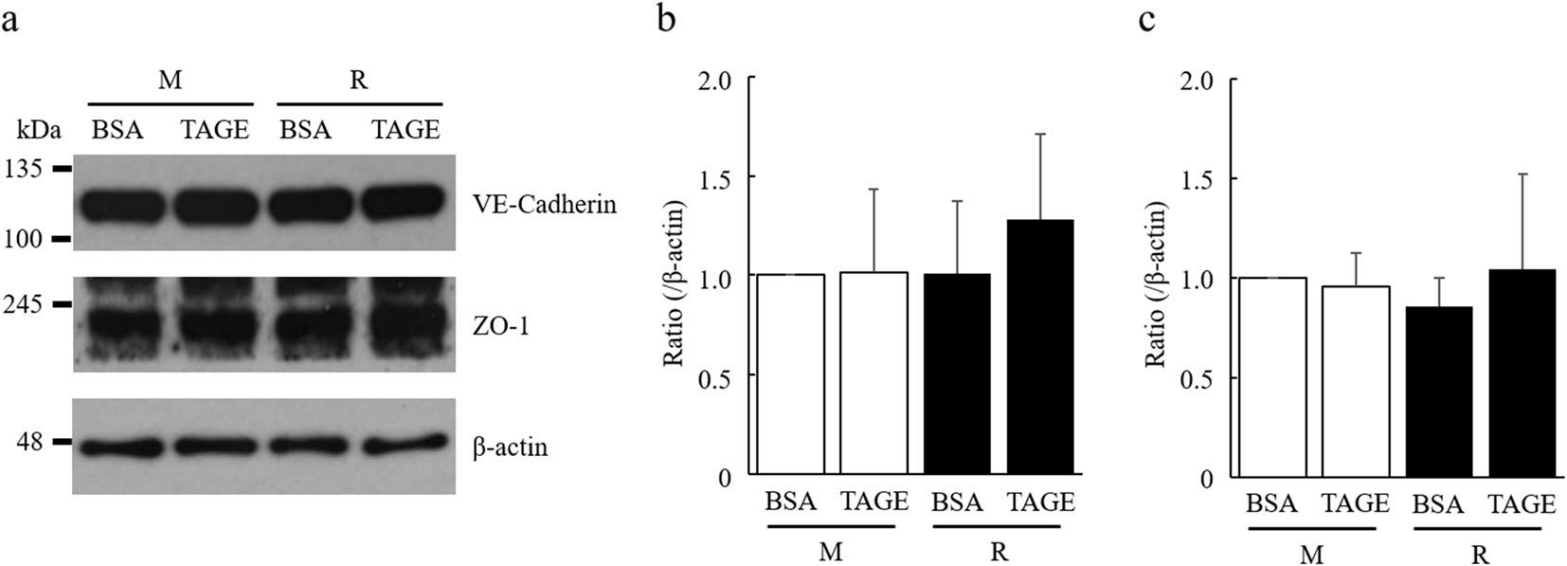
Expression of VE-cadherin and ZO-1 proteins by TAGE. (**a**) VE-cadherin (approximately 130 kDa), ZO-1 (approximately 220 kDa) and β-actin (approximately 48 kDa) proteins were detected using western blotting. Size markers (kDa) are shown on the left. (**b**) Densitometry analysis for VE-cadherin protein. (**c**) Densitometry analysis for ZO-1 protein. *M* mock cells, *R* RasGRP2-stable overexpression cells. Data shown as the mean ± SD (n = 3).

**Figure 5:**
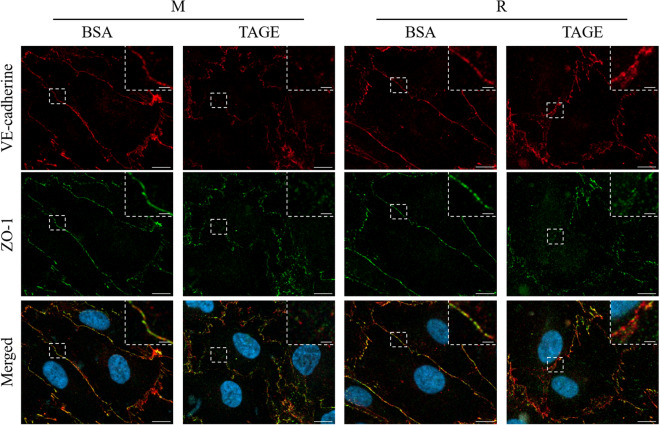
Protective effect of RasGRP2 against TAGE-induced VE-cadherin perturbation. VE-cadherin and ZO-1 proteins were stained and visualized using confocal microscopy. *M* mock cells, *R* RasGRP2-stable overexpression cells. VE-cadherin (red), ZO-1 (green), and merged, including nucleus (blue), images are shown. The dashed box indicates the enlarged area, which is shown in the upper right corner. Scale bar, 10 μm. An enlarged area is shown for each image with a scale bar of 2 μm.

We examined the influence of TAGE treatment on both vascular endothelial (VE)-cadherin proteins that constitute adherens junctions (AJs) and zonula occludens-1 (ZO-1) proteins that comprise tight junctions (TJs). The expression levels of VE-cadherin and ZO-1 were not reduced by TAGE treatment in both M and R cells (Fig. 4a–c). The localization of these proteins was estimated by immunofluorescence staining. When treated with BSA, these proteins were localized at intercellular adhesion sites in both M and R cells. The localization of VE-cadherin was perturbed by TAGE treatment in M cells, but not in R cells. On the other hand, the localization of ZO-1 was perturbed by TAGE treatment in both M and R cells (Fig. 5). These results suggest that RasGRP2 influences the stabilization of AJs.

**Figure 6:**
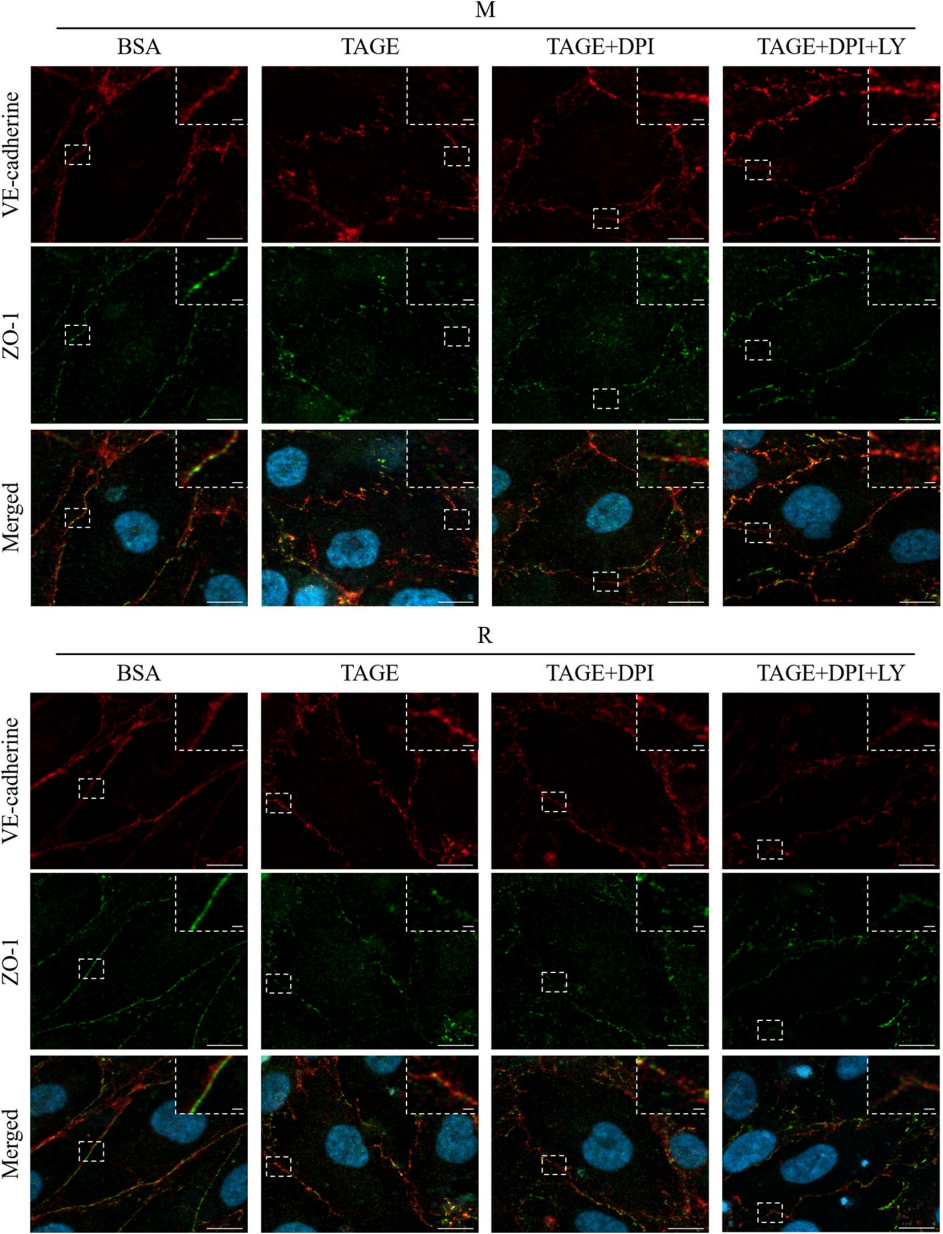
Protection of TAGE-induced VE-cadherin perturbation via ROS and non-ROS pathways by RasGRP2. DPI, an inhibitor of NOX, and LY, an inhibitor of the PI3K-Akt pathway, were used to inhibit TAGE-induced ROS production via NOX and RasGRP2-induced Akt activation, respectively. VE-cadherin and ZO-1 proteins were stained and visualized using confocal microscopy. *M* mock cells, *R* RasGRP2-stable overexpression cells, *DPI* diphenyleneiodonium, *LY* LY294002. VE-cadherin (red), ZO-1 (green), and merged, including nucleus (blue), images are shown. The dashed box indicates the enlarged area, which is shown in the upper right corner. Scale bar, 10 μm. An enlarged area is shown for each image with a scale bar of 2 μm.

Furthermore, in the inhibitor experiment, the TAGE-induced perturbation of VE-cadherin in M cells was protected by DPI pretreatment, but the perturbation of ZO-1 was not protected. Similar results were obtained for the DPI + LY co-pretreatment. On the other hand, in R cells, no change due to DPI pretreatment was observed, but DPI + LY co-treatment showed a slight perturbation of VE-cadherin compared to TAGE treatment alone (Fig. 6). These results indicate that RasGRP2 protects TAGE-induced perturbation of VE-cadherin via the ROS pathway and non-ROS pathway.

### RasGRP2 suppresses TAGE-induced vascular hyper-permeability through the Rap1 and R-Ras pathways

**Figure 7:**
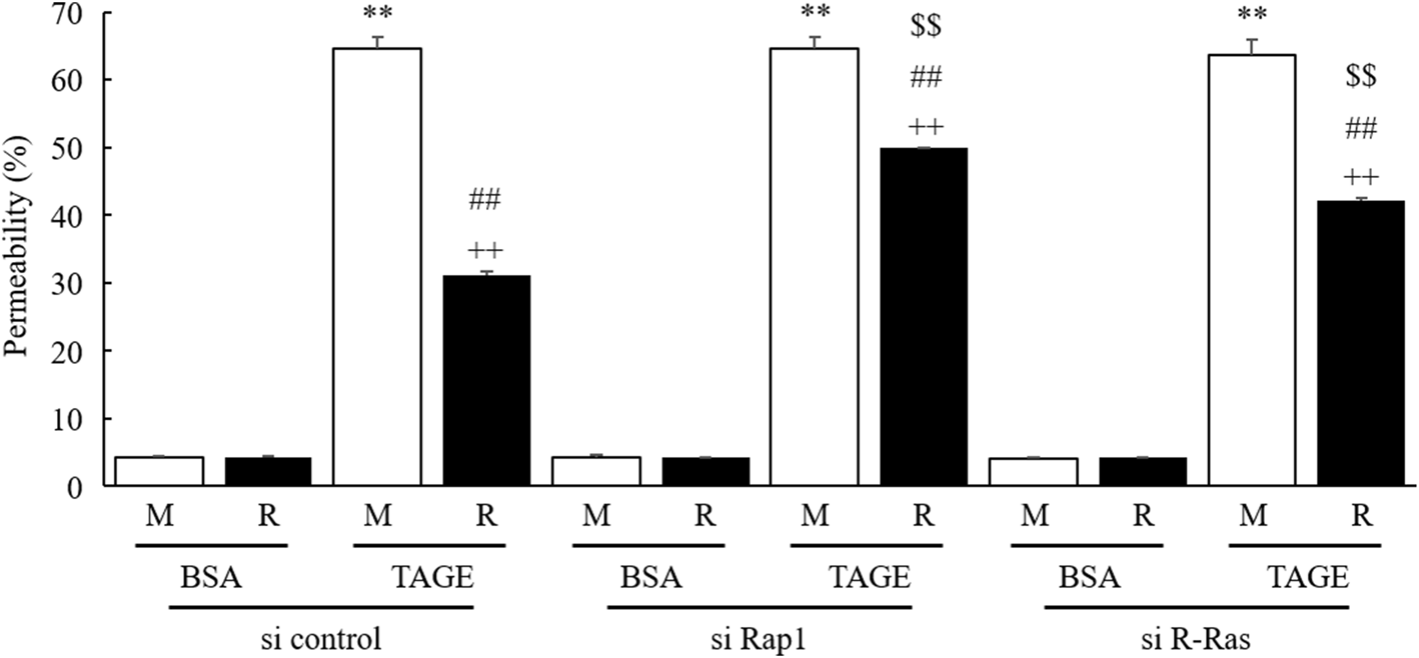
RasGRP2 via Rap1 and R-Ras pathways protects against TAGE-induced vascular hyper-permeability. Cells were treated with siRNAs against Rap1, R-Ras or negative control siRNA. Permeability was measured using FITC-Dextran. *M* mock cells, *R* RasGRP2-stable overexpression cells. Data shown as the mean ± SD (n = 3), **P < 0.01 compared with BSA-treated M cells, ^++^*P* < 0.01 compared with BSA-treated R cells, ^##^*P* < 0.01 compared with each TAGE-treated M cells, and ^$$^*P* < 0.01 compared with each si control-treated cells.

To investigate the suppression pathway of RasGRP2 on TAGE-induced hyper-permeability in vascular endothelial cells, we compared the vascular permeability of M and R cells by Rap1 and R-Ras knockdown. We confirmed that Rap1 and R-Ras proteins were already reduced 24 h after each 90 nM siRNA treatment compared to si control (Fig. S4). In M cells, no further increase in TAGE-induced hyper-permeability was observed in Rap1 and R-Ras knockdown compared to the si control. On the other hand, in R cells, TAGE-induced hyper-permeability was significantly increased by the knockdown of Rap1 and R-Ras (Fig. 7). This result indicates that the suppression of vascular hyper-permeability by RasGRP2 depends on both pathways.

## Discussion

Vascular endothelial cells form a monolayer inside the blood vessels and function as a barrier to regulate blood components permeation through the vessel wall. The endothelial barrier's permeability is regulated by various cell–cell junctions comprising AJs, TJs, and gap junctions. Disruption of AJs or TJs can cause various diseases, such as DR^25^. In the vascular endothelium, AJs consist of VE-cadherin and its associated α-, β-, and p120-catenin adhesion complexes, and TJs consist of occludin, claudins, junctional adhesion molecules, and associated ZO-1, -2, and -3 proteins^25,26^. Herein, we demonstrated that TAGE enhances permeability in the vascular endothelium by disrupting both VE-cadherins that constitute AJs, and ZO-1s, constituting TJs (Figs. 1, 2, and 5). Furthermore, we previously reported that TAGE causes apoptosis of retinal pericytes that surround the endothelial tube's extraluminal surface, resulting in enhanced vascular permeability^27^. These results suggest the involvement of TAGE in the pathogenesis of DR.

AGEs are known to enhance vascular permeability, and several mechanisms have been proposed. Navaratna et al. reported that AGEs degrade VE-cadherin by activating matrix metalloproteinase (MMP)-2 and MMP-9^28,29^, while Otero et al. also noted that ROS production by AGEs causes decreased expression of VE-cadherin, β-catenin, and γ-catenin, thereby enhancing vascular permeability^30^. However, Hirose et al. reported that actin stress fiber increase, via the AGE-Rho pathway, can cause vascular permeability without changing the expression of VE-cadherin^31^. Furthermore, it has been reported that an AGE-dependent increase in F-actin, via the Rho-pMLC pathway, also causes permeability^32–34^. Recent studies have shown that phosphorylation of Src by AGE-induced, NOX-mediated ROS production enhances vascular permeability through the phosphorylation of VE-cadherin^35,36^. AGE-induced ROS production also enhances vascular permeability by reducing ZO-1 expression via the downregulation of actin-depolymerizing factors^37^. These reports, taken together, show that AGEs disrupt both AJs and TJs in complex pathways. Indeed, we have shown that TAGEs enhance vascular permeability through ROS and non-ROS pathways without decreasing cell viability (Figs. 2 and 3). Although VE-cadherin and ZO-1 protein expression was not decreased in TAGE-induced vascular hyper-permeability (Fig. 4), the localization of these proteins to the intercellular adhesion sites was perturbed through ROS and non-ROS pathways (Fig. 6). The disruption of these protein localizations may be due to phosphorylation by TAGE. Although phosphorylation of ZO-1 is not a mechanism of AGE-induced permeability, it has recently been reported as a cause of vascular hyper-permeability^38,39^. This finding suggests that TAGE causes vascular hyper-permeability by disrupting AJs and TJs through both ROS and non-ROS pathways.

We have previously reported that RasGRP2 inhibits NOX via Rap1 and activates Akt via R-Ras in vascular endothelial cells^23,24^. Rap1 is activated by RasGRP2 and other GEFs, such as EPAC, PDZ-GEF, and C3G. Cullere et al. reported that EPAC-activated Rap1 had enhanced endothelial barrier function via its effector, AF-6, and offsets thrombin-induced Rho-dependent vascular permeability^40^. Pannekoek et al. reported that PDZ-GEF-activated Rap1 stabilizes cell–cell junctions and controls basal barrier function^41^. Birukova et al. reported that C3G-activated Rap1 promotes endothelial barrier repair after thrombin treatment by downregulating the Rho pathway^42^. This finding implies that the role of Rap1 in vascular permeability differs depending on the associated GEF. Indeed, we found that activation of Rap1 by RasGRP2 suppressed TAGE-induced vascular hyper-permeability by suppressing NOX-mediated ROS production without enhancing basal barrier function (Figs. 1, 3, and 7). Furthermore, although the GEF of R-Ras in the vascular endothelium has not been well investigated, Griffiths et al. demonstrated that siRNA-knockdown of R-Ras in vascular endothelial cells reduces the associations of R-Ras and Filamin A. This indicates an increase in the phosphorylation of VE-cadherin Tyr731 and, as a result, promotes vascular permeability^43^. We also found that activation of R-Ras by RasGRP2 suppressed TAGE-induced vascular hyper-permeability via the non-ROS pathway (Figs. 3 and 7). These results suggest that RasGRP2 can suppress multiple vascular hyper-permeability signals via activation of both pathways. It was also shown that RasGRP2 suppression of TAGE-induced vascular hyper-permeability is due to the protection of VE-cadherin protein perturbation (Figs. 5 and 6), suggesting that RasGRP2 protects TAGE-induced disruption of AJs rather than TJs.

**Figure 8:**
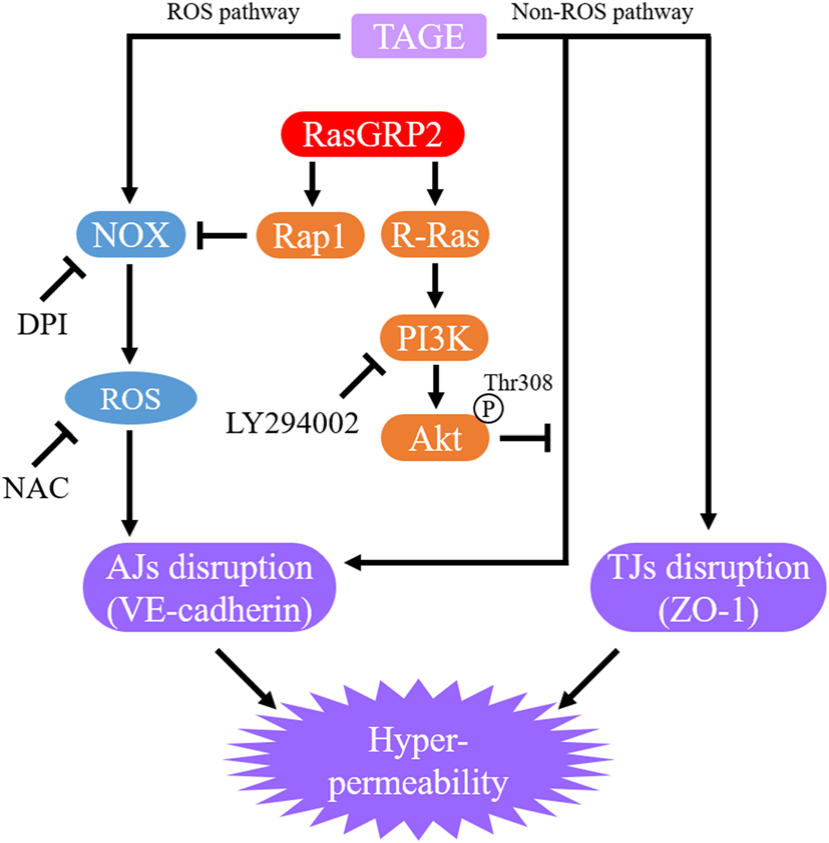
Proposed model for TAGE-induced vascular hyper-permeability suppression via the Rap1 and R-Ras pathways by RasGRP2. *TAGE* glyceraldehyde-derived toxic advanced glycation end-products, *RasGRP2* ras guanyl nucleotide releasing protein 2, *NOX* NADPH oxidase, *ROS* reactive oxygen species, *PI3K* phosphoinositide 3-kinase, *AJs* adherens junctions, *TJs* tight junctions, *VE-cadherin* vascular endothelial-cadherin, *ZO-1* zonula occludens-1, *Thr* threonine, *P* phosphorylation.

In this study, we demonstrated that TAGE enhances vascular permeability by disrupting AJs and TJs via complex routes, such as ROS and non-ROS pathways, and that RasGRP2, via the Rap1 and R-Ras pathways, suppresses vascular hyper-permeability by protecting AJ disruption (Fig. 8). Therefore, RasGRP2, which activates both Rap1 and R-Ras, may be an important protective factor against vascular permeability. Compounds that modulate RasGRP2 expression and activity may help develop new therapeutic strategies for AGE-induced DR. Further investigation of RasGRP2 expression mechanisms and the activity regulation of RasGRP2 is necessary.

## Methods

### Reagents

All reagents were commercially available, of high purity, and used according to protocol. TAGE and BSA were prepared as previously described^44^. Briefly, 25 mg/mL of bovine serum albumin (BSA, A0281; Sigma-Aldrich) was incubated at 37 ℃ for seven days under sterile conditions with 0.1 mol/L glyceraldehyde (17014-94; Nacalai Tesque Inc.) and 5 mmol/L diethylenetriaminepentaacetic acid (D022; Dojindo Laboratories) in 0.2 mol/L phosphate buffer (pH 7.4). As a control, unglycated BSA was incubated under the same conditions but without glyceraldehyde. The unglycated and glycated albumins were purified using a PD-10 column (17085101; GE Healthcare) and dialyzed against PBS. All preparations were tested for endotoxin using the Endospecy ES-24S set (020170; Seikagaku Co.). Protein concentrations were determined using the Dc protein assay reagent (5000111JA; Bio-Rad Laboratories) using BSA as a standard. NAC (017-05131) was purchased from FUJIFILM Wako Pure Chemical Corporation, and DPI (81050), apocynin (11976), and LY (70920) were purchased from Cayman Chemical.

### Cell cultures

The TERT HUVEC were kindly provided by Dr. Kazuto Nishio (Kindai University). HUVEC (C-12203; PromoCell) and TERT HUVEC were grown in endothelial cell growth medium (C-22010; PromoCell) under standard cell culture conditions (humidified atmosphere, 5% CO_2_, 37 °C). Stable cell lines were prepared as previously described^23^. Briefly, the DNA fragment of *rasgrp2* was amplified from human placenta cDNA (K1420-1; Clontech) using the Expand Long Range dNTPack (4829042001; Sigma-Aldrich), pEB Multi-Hyg (050-08121; FUJIFILM Wako Pure Chemical Corporation) was used as a vector, and ViaFect Transfection Reagent (E4981; Promega) was used as the transfection reagent. Cells were seeded in a 24-well plate, grown overnight, transfected, and purified to prepare TERT HUVEC R and mock cell lines. Transfected cells were purified with 50 μg/mL hygromycin B solution (09287-84; Nacalai Tesque Inc.).

Cells were then seeded (0.6 × 10^5^ cells/mL) in culture dishes or cell culture inserts and incubated for six days (medium changed every second day) before experiments. TEER measurements on barrier formation were carried out during this time. Cells were pre-incubated with or without 5 mM NAC, 20 μM DPI, 30 μM apocynin, or 10 μM LY for 2 h, and incubated with 50 μg/mL BSA or TAGE for 24–48 h to investigate the specific effects of RasGRP2.

### Measurement of TEER and permeability

The upper and bottom chambers were filled with 250 μL and 800 μL of the medium, respectively, and cells were cultured as a monolayer on a cell culture insert (pore size 0.4 µm, high density, 353495; Corning Falcon). Values were recorded using Millicell-ERS (MERS00002; Merck Millipore), and the TEER value was obtained by subtracting the resistance of the corresponding naked filter from that of the cell monolayer filter.

For the permeability measurements, the culture medium was removed from the upper and bottom chambers, the upper chamber was replaced with 150 μL FITC-Dextran (1 mg/mL, MW 70000, 90718-1G; Sigma-Aldrich) diluted with medium, and the bottom chamber was replaced with 500 μL medium. After 20 min, 100 μL of the bottom chamber medium was sampled. The fluorescence of FITC-Dextran was measured using a multilabel counter (ARVO MX-1 1420; Perkin Elmer), and excitation/emission wavelengths were set at 485/535 nm. The fluorescence value of FITC-Dextran that passed through the cell culture insert without cells was shown to be 100%.

### Cell viability

Cell viability was determined as previously described^24^. Briefly, after 48 h incubation with BSA or TAGE, the cells were incubated with 10 µL/well of Cell Counting Kit-8 assay solution (CK04; Dojindo Laboratories) for 2 h. The net difference in absorbance (A450–A650) was used as a measure of cell viability. The viability of BSA-treated M cells was taken as 100%.

### Measurement of intracellular ROS

Intracellular ROS was measured as previously described^24^. Briefly, after 24 h incubation with BSA or TAGE, the cells were incubated with 5 μM CellROX Green (C10444; Thermo Fisher Scientific) for 1 h. Then, the medium was exchanged, and the fluorescence image was acquired. The fluorescence area was calculated using the average value of three randomly selected fields. The ROS ratio in TAGE-treated M cells was 1.

### Preparation of cell lysate and western blotting

After 48 h incubation with BSA or TAGE, preparation of cell lysate and western blot analysis were performed as previously described^24^, using the following antibodies: anti-VE-cadherin (sc-9989 at 1:5000; Santa Cruz Biotechnology, Inc.), rabbit anti-ZO-1 (GTX108627 at 1:1500; GeneTex), mouse anti-β-actin (sc-47778 at 1:12,000; Santa Cruz Biotechnology, Inc.), anti-rabbit IgG (GTX77057 at 1:5000; GeneTex), and anti-mouse IgG (P0260 at 1:5000, Dako Denmark A/S).

### Immunofluorescence staining

After the cells were incubated with BSA or TAGE for 48 h, immunofluorescence staining was performed as previously described^45^. Cells grown on a Nunc Lab-Tek II chamber slide (154534PK; Thermo Fisher Scientific) were removed from the medium and fixed in 95% ethanol for 1 h at room temperature. Cells were rinsed with PBS (−) and incubated with 0.5% Triton X-100 in PBS (−) for 5 min at room temperature. The cells were then blocked for 5 min using Blocking One Histo (06349-64; Nacalai Tesque Inc.) before being incubated with mouse anti-VE-cadherin (sc-9989 at 1:2000; Santa Cruz Biotechnology, Inc.) or rabbit anti-ZO-1 (GTX108627 at 1:1000; GeneTex) antibody in Can Get Signal Immunostain Immunoreaction Enhancer Solution B (NKB-601; Toyobo) for 1 h. Subsequently, the cells were rinsed three times with PBS-T and incubated with Cy5 conjugated goat anti-mouse IgG (AP200S at 1:1000; Chemicon International) or Cy3 conjugated goat anti-rabbit IgG (AP132C at 1:2000; Chemicon International) in Can Get Signal Immunostain Immunoreaction Enhancer Solution B for 1 h. After rinsing three times with PBS-T, the coverslips were mounted on glass slides using a ProLong Glass Antifade Mountant with NucBlue (P36983; Thermo Fisher Scientific), and confocal microscopy was performed using a scanning confocal microscope system (LSM 800; Carl Zeiss).

### Knockdown by siRNA

Rap1 siRNA (90 nM, SASI_Hs01_00040403; Sigma-Aldrich), R-Ras siRNAs (90 nM, siRNA mix, EHU0225113; Sigma-Aldrich), or negative control siRNA (90 nM, SIC-001; Sigma-Aldrich) were transfected into cells according to the manufacturer's instructions. Briefly, cells were incubated in cell culture inserts for four days (medium changed every second day). The medium was changed to 10% FCS Endothelial Cell Growth Medium 2 (C-22211; PromoCell) without heparin (10% EGM-2/heparin(−)). Then, a mixture of MISSION siRNA Transfection Reagent (S1452; Sigma-Aldrich) and siRNA was pre-incubated for 10 min, and then transfected. Two days later (6th day after starting cultivation), the medium was changed to 10% EGM-2/heparin(−) and siRNA transfection, and BSA and TAGE treatment were performed.

### Statistical analysis

All experiments were performed in duplicate and repeated at least two or three times; each experiment yielded essentially identical results. Data are expressed as mean ± standard deviation (SD). The significance of difference between group means was determined using one-way analysis of variance and t-test. P < 0.05 was considered statistically significant.

## Supplementary Information

Supplementary Figures.

## Data Availability

The datasets generated and analyzed during the current study are available from the corresponding author upon reasonable request.
