# Supplementary material for: RasGRP2 inhibits glyceraldehyde-derived toxic advanced glycation end-products from inducing permeability in vascular endothelial cells
Source: Sci Rep. 2021 Feb 3;11:2959. doi: 10.1038/s41598-021-82619-0 (PMC7859393; doi:10.1038/s41598-021-82619-0)

Supplementary Information

RasGRP2 inhibits glyceraldehyde-derived toxic advanced glycation end-products from inducing permeability in vascular endothelial cells

Jun-ichi Takino[1*], Takuma Sato[1], Takumi Kanetaka[1], Kasumi Okihara[1], Kentaro Nagamine[2], Masayoshi Takeuchi[3], and Takamitsu Hori[1]

[1] Faculty of Pharmaceutical Sciences, Hiroshima International University, Hiroshima, Japan

[2] Faculty of Health Sciences, Hiroshima International University, Hiroshima, Japan

[3] Department of Advanced Medicine, Kanazawa Medical University, Ishikawa, Japan

*To whom correspondence should be addressed: Jun-ichi Takino: j-takino@hirokoku-u.ac.jp

Included in Supplementary Information:

Supplemental Figures 1-5

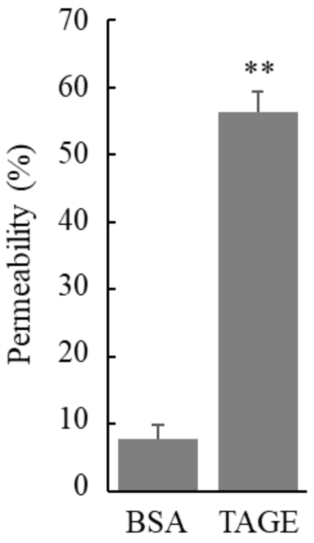

Figure S1. Induction of vascular hyper-permeability by TAGE in HUVEC. Permeability was measured using FITC-Dextran. Data shown as the mean ± SD (n = 3), ***P* < 0.01 compared with BSA-treated cells.

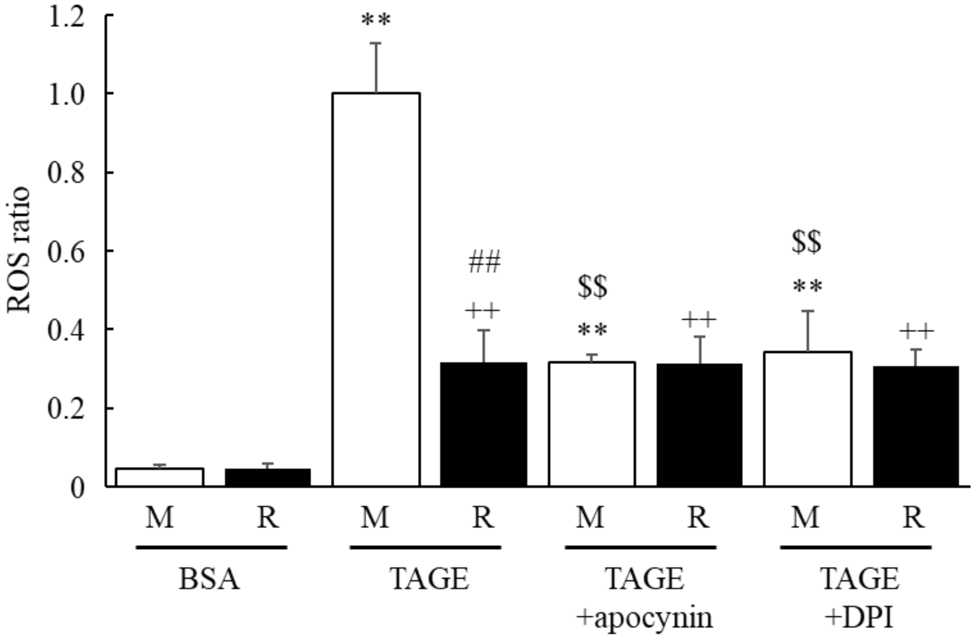

Figure S2. Suppression of TAGE-induced NOX-dependent ROS production by RasGRP2. Intracellular ROS was determined by CellROX Green. M: mock cells, R: RasGRP2-stable overexpression cells, DPI: diphenyleneiodonium. Data shown as the mean ± SD (n = 3), ***P* < 0.01 compared with BSA-treated M cells, $^{++}P < 0.01$ compared with BSA-treated R cells, $^{\#\#}P < 0.01$ compared with each TAGE-treated M cells, and $^{\$\$}P < 0.01$ compared with TAGE-treated cells alone.

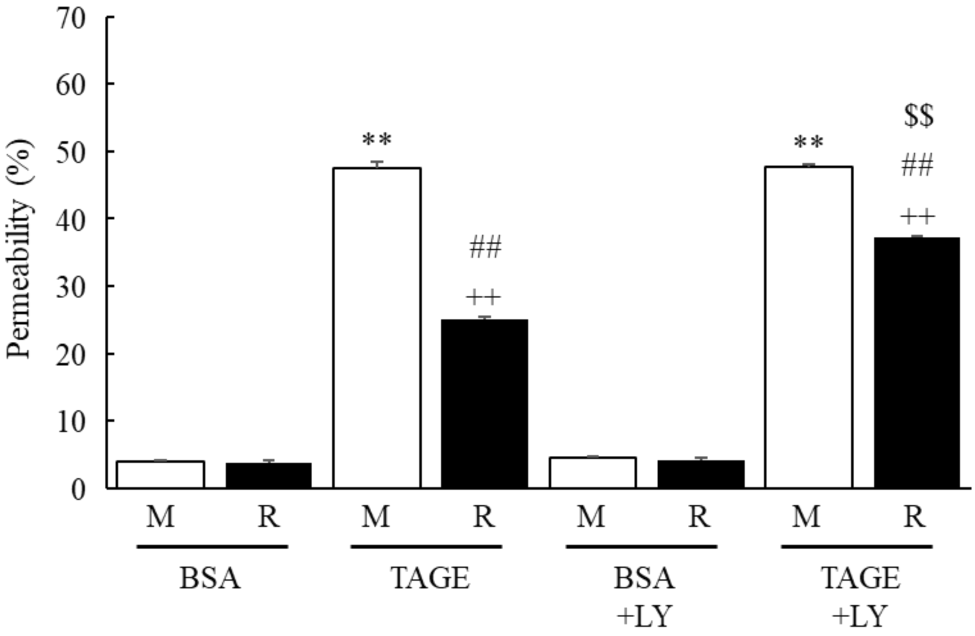

Figure S3. Suppression of TAGE-induced ROS-independent vascular hyper-permeability by RasGRP2. Permeability was measured using FITC-Dextran. M: mock cells, R: RasGRP2-stable overexpression cells, LY: LY294002. Data shown as the mean ± SD (n = 3), $^{**}P < 0.01$ compared with BSA-treated M cells, $^{++}P < 0.01$ compared with BSA-treated R cells, $^{\#\#}P < 0.01$ compared with each TAGE-treated M cells, and $^{\$\$}P < 0.01$ compared with TAGE-treated cells alone.

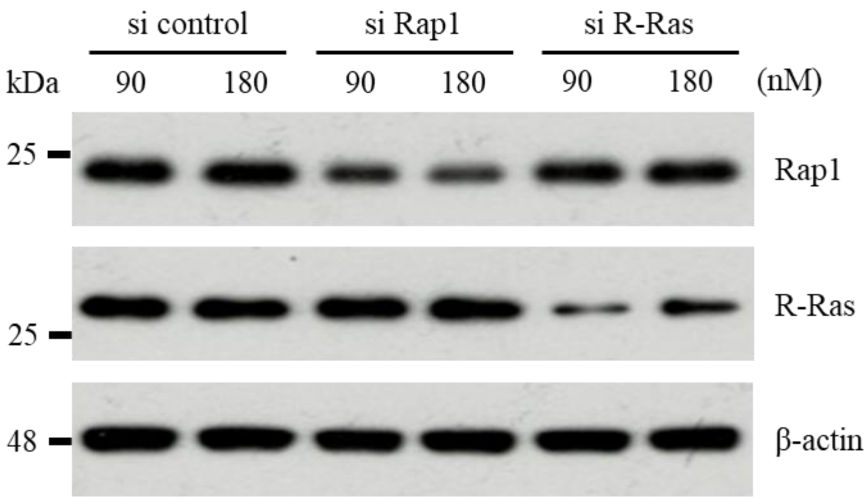

Figure S4. The knockdown efficiency of siRap1 and siR-Ras. M cells were treated with siRNAs against Rap1, R-Ras or negative control siRNA. After 24 h, Rap1 (approximately 22 kDa), R-Ras (approximately 25 kDa) and β-actin (approximately 48 kDa) proteins were detected using western blotting. Size markers (kDa) are shown on the left.

Figure S5. Unprocessed blot images

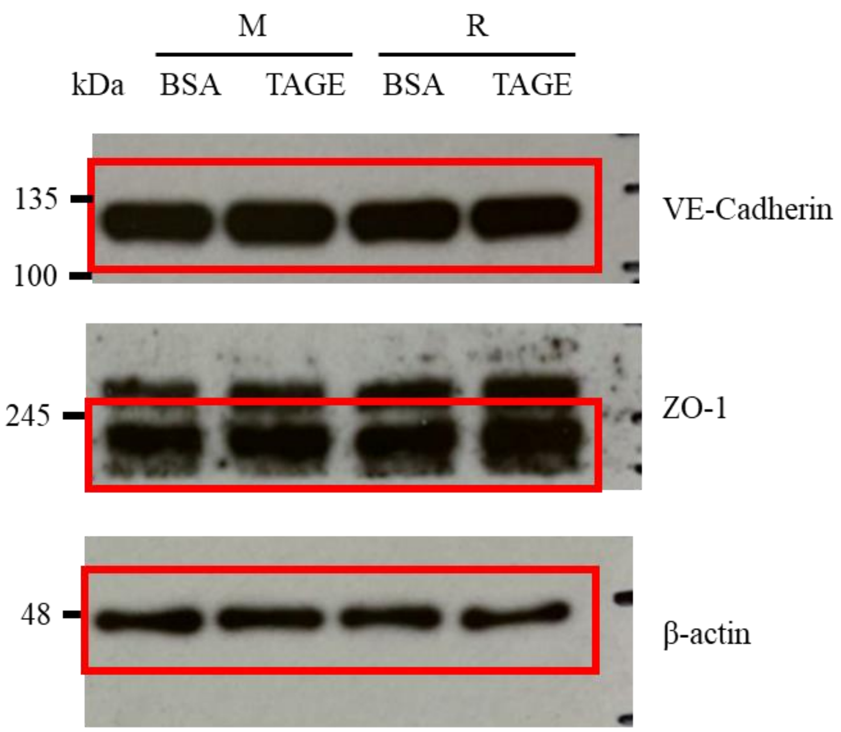

Fig. 4a

Figure S5. Unprocessed blot images (Continued)

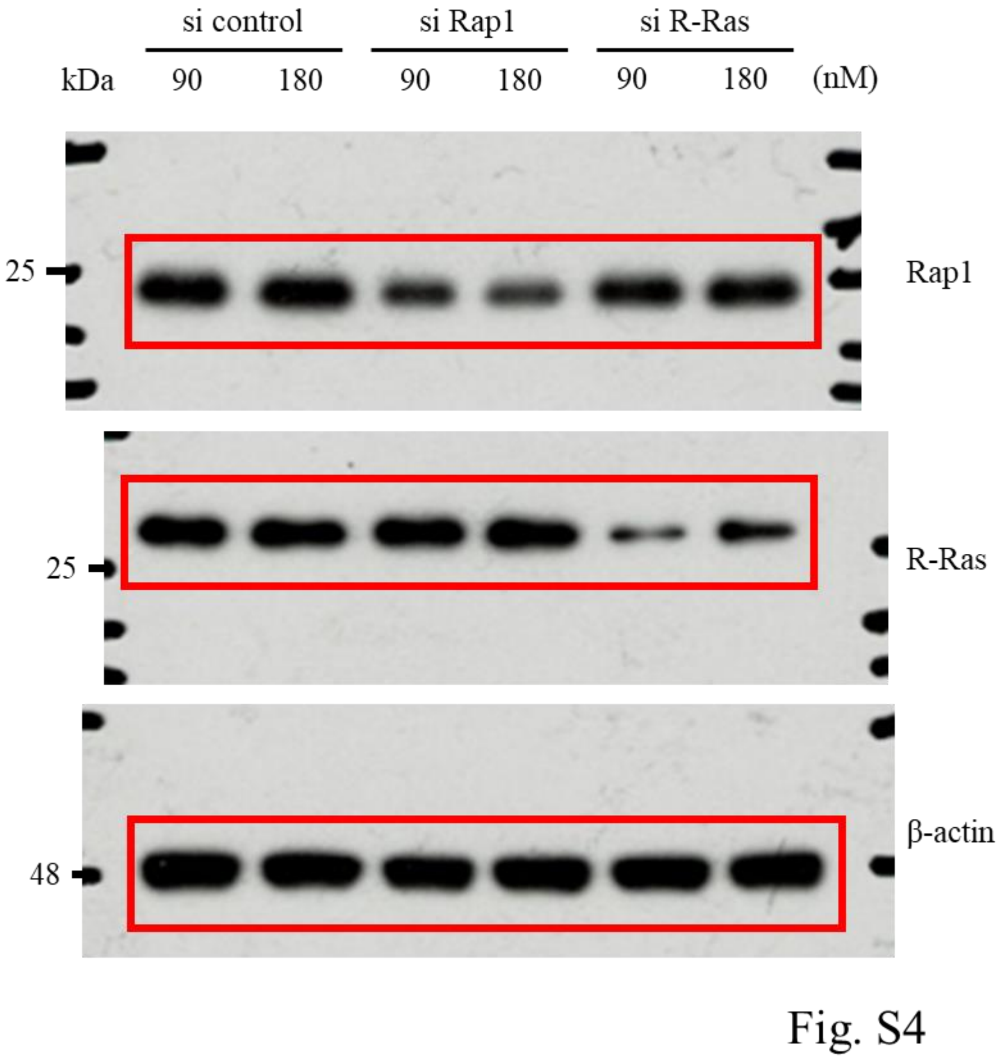

Supplement: Supplementary file 1 — Supplementary Figures. [file 41598_2021_82619_MOESM1_ESM.pdf]
